# Environmentally Friendly UV-Protective Polyacrylate/TiO_2_ Nanocoatings

**DOI:** 10.3390/polym13162609

**Published:** 2021-08-05

**Authors:** Martina Zeljko, Vesna Ocelić Bulatović, Vedrana Špada, Sanja Lučić Blagojević

**Affiliations:** 1 Faculty of Chemical Engineering and Technology, University of Zagreb, 10000 Zagreb, Croatia; 2 Faculty of Metallurgy, University of Zagreb, 44000 Sisak, Croatia; vocelicbu@simet.hr; 3 METRIS Materials Research Centre of Region of Istria, 52100 Pula, Croatia; vspada@iv.hr

**Keywords:** wood nanocoatings, UV protection, polyacrylate, rutile TiO_2_

## Abstract

The development of coatings that maintain the attractive natural appearance of wood while providing ultraviolet (UV) protection is extremely important for the widespread use of wood products. In this study, the influence of different types (powder form and aqueous dispersions) of TiO_2_ in an amount of 1.0 wt% by monomer weight on the properties of environmentally friendly polyacrylate (PA)/TiO_2_ emulsions prepared by ex situ and in situ polymerization, as well as on the UV-protective properties of the coating films, was investigated. The results showed that the addition of TiO_2_ significantly affected the particle size distribution of PA and the viscosity of PA varied according to the preparation method. Compared with the ex situ preparation method, in situ polymerization provides better dispersibility of TiO_2_ nanoparticles in PA coating film, as well as a better UV protection effect and greater transparency of the coating films. Better morphology and transparency of nanocoating films were achieved by adding TiO_2_ nanofillers in aqueous dispersion as compared to the addition of TiO_2_ in powder form. An increase in the glass transition temperature during UV exposure associated with cross-linking in the polymer was less pronounced in the in situ-prepared coating films, confirming better UV protection, while the photocatalytic effect of TiO_2_ was more pronounced in the ex situ-prepared coating films. The results indicate that the method of preparation has a significant influence on the properties of the coating films.

## 1. Introduction

Materials, such as wood, without adequate protection against weathering are subject to photochemical reactions that lead to significant changes, mainly affecting the wood surface, which significantly affects the esthetic appearance and performance of wood (change in color, gloss, brightness, roughness). The exposure of wood to sunlight, i.e., UV radiation (ultraviolet radiation), and water is the main factor of wood weathering. UV radiation affects the color of wood and causes it to turn gray. Prolonged irradiation weakens the surface fibers due to the depolymerization of lignin and carbohydrates in the cell walls of wood, resulting in mold growth, cracking, splitting, and warping of the wood surface [1]. UV radiation belongs to the non-ionizing region of the electromagnetic spectrum, which comprises about 8–9% of the total solar radiation [2]. Various polymer coatings are used to protect materials from external influences such as UV radiation.

Polyacrylate-based coatings are widely used for interior and exterior applications due to their good resistance to UV light, wear resistance and good elasticity with sufficient chemical stability [3]. They have the great advantage that they can be produced as aqueous emulsions, thus providing an environmentally friendly alternative to organic solvent-based coatings [3]. Polyacrylate coatings are largely obtained by radical polymerization of acrylate and methacrylate monomers [4]. From an industrial point of view, which includes aqueous polymers prepared by emulsion polymerization, it is witnessing steady growth due to its ability to control the properties of emulsion polymers.

One of the main disadvantages of using polymers as coatings that are used outdoors is accelerated degradation due to environmental conditions. UV exposure causes photooxidative degradation, leading to polymer chain rupture, radical formation, and molecular weight reduction. These changes lead to the deterioration of mechanical properties and consequently inadequate further UV protection. Previous studies [5] have shown that polyacrylates are affected by UV exposure as chain scission and cross-linking reactions occur. Soeriyadia et al. [6] studied acrylate and methacrylate degradation of polymers and concluded that one mechanism of degradation involves the loss of ester side groups and the formation of methacrylic acid and, at the end, cross-linking.

Polymer coatings such as polyacrylate can be protected from harmful UV radiation by the addition of pigments, UV absorbers and/or radical scavengers [7,8,9]. Due to the strong absorptivity of UV light, inorganic particles such as TiO_2_ [8,9,10,11] and ZnO [12,13] are usually added to the polymer matrix to provide UV-shielding properties. The key aspect is to disperse the TiO_2_ nanoparticles homogeneously in the polymer matrix to obtain the desired properties. However, due to the high surface energy, TiO_2_ nanoparticles tend to agglomerate in the polymer matrix, which makes dispersion a difficult task, especially at higher loadings [14]. Based on the crystal structures, there are three forms of nano TiO_2_: anatase, rutile, and brookite. Brookite is unstable and therefore not suitable for commercial use. For UV protection of coatings, rutile TiO_2_ is more suitable as it is less photoactive than anatase [15,16,17] because the band gap of rutile (3.0 eV) is smaller than that of anatase (3.2 eV). The smaller band gap of rutile TiO_2_ gives it greater reducibility and the ability to generate more photoelectrons, resulting in higher UV absorption [18]. TiO_2_ nanoparticles in a rutile crystal structure provide protection below 400 nm by UV light absorption and above 400 nm by light scattering. Moreover, a major advantage of TiO_2_ nanoparticles over organic UV absorbers is that they do not migrate and remain dispersed in the polymer material [19].

Various methods such as mechanical mixing, the sol–gel technique and in situ polymerization have already been developed to prepare TiO_2_/polymer nanocomposites [20]. Man et al. [21] have shown that nanocomposites prepared by emulsion polymerization in the presence of TiO_2_ nanoparticles (in situ synthesis) lead to better particle distribution within the polymer matrix and strong chemical bonds between TiO_2_ and the polymer, compared to the mechanical mixing of TiO_2_ nanoparticles and PA emulsion (ex situ synthesis).

The performance of UV-protective coatings with inorganic nanoparticles as UV absorbers has been studied by many authors [13,22,23,24,25]. Nguyen et al. [13] found that the presence of rutile TiO_2_ in the acrylate nanocomposite reduced the transmittance in the UV and visible region and retarded the UV degradation of the acrylate coating. In another study by Nguyen et al. [22], the results of FTIR analysis showed that the presence of TiO_2_ leads to a slowing down of the formation of oxidative products during the aging process as TiO_2_ nanoparticles retard UV degradation. Antune et al. [23] investigated the effect of TiO_2_ on the UV degradation and thermal stability of poly(3-hydroxybutyrate-co-3-hydroxyvalerate) (PHBV) and suggested that the addition of TiO_2_ retards the process of UV degradation and thermal degradation of PHBV by limiting the physical mobility of the polymer chains. Yang et al. [24] reported that the addition of TiO_2_ to PVC effectively inhibits photooxidation and chain scission, and improves the weatherability of PVC so that the mechanical properties are maintained during outdoor weathering. Coatings with TiO_2_ have high absorption in the UV range, but also in the visible part of the spectrum (400–800 nm), which affects the transparency of the coating and causes surface heating, as indicated by Aloui et al. [25].

In this work, polyacrylate PA/TiO_2_ nanocoating films were prepared by ex situ and in situ emulsion polymerization. The aim was to prepare environmentally friendly coating films with good UV shielding ability and high transparency and to investigate the effects of nano TiO_2_ and UV exposure on the properties of the PA coating. Considering the considerable number of different TiO_2_ types on the market, this work investigated the influence of the different TiO_2_ types on the properties of PA as well as the influence of the preparation methods (in situ and ex situ) on the properties of the coating films.

## 2. Materials and Methods

### 2.1. Materials

The monomers methyl methacrylate (MMA) and butyl acrylate (BA), the initiator ammonium persulfate (APS) supplied by Acros Organics (Geel, Belgium) and the anionic emulsifier sodium dodecyl sulfate (SDS) (DISPONIL FES) donated by BASF (Ludwigschafen, Germany) were used for emulsion polymerization. Four types of TiO_2_ nanofillers, two in powder form (PN and PM PVP) and two in aqueous dispersion form (DN and DW), provided by US Research Nanomaterials (Houston, TX, USA) were used for emulsion polymerization. The average particle size of all nanofillers is 30 nm. PM PVP is modified with polyvinylpyrrolidone (PVP), while the PN nanopowder is not. DN aqueous dispersion is produced in large plants and the particle size range is wide (from 10–120 nm), while DW is produced in a laboratory, involves an additional process of centrifugation and TiO_2_ is completely dispersed in water. The properties of the nanofillers are summarized in Table 1.

### 2.2. Sample Preparation

**Figure 1:**
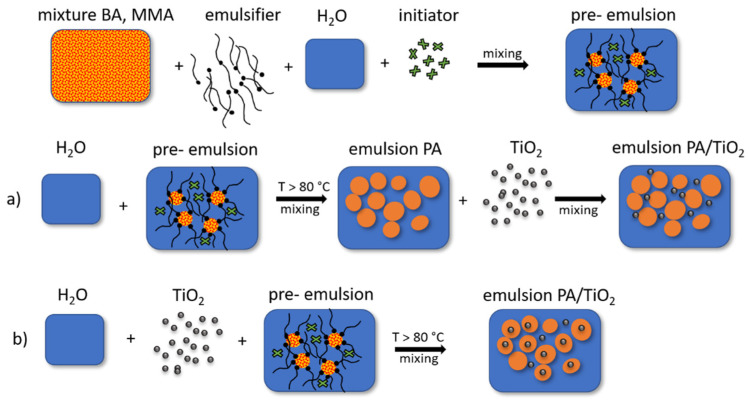
Schemes of (**a**) ex situ and (**b**) in situ preparation of PA/TiO_2_ emulsions.

Emulsions were prepared by ex situ and in situ emulsion polymerization, as schematically shown in Figure 1. Emulsions were synthesized with concentrations of 1.0 wt% TiO_2_ by monomer weight.

Neat PA emulsions were prepared by the following procedure. The 1000 mL reactor equipped with a cooler, dropping funnel, and mechanical stirrer was filled with deionized water and heated to 82 °C. The pre-emulsion was prepared from monomers, a portion of the initiator ammonium persulfate (APS) (0.18%) and the anionic emulsifier sodium dodecyl sulfate (SDS) (11.10%) and then added dropwise to the reactor. At the end of this phase, a small amount of initiator APS was added to complete the polymerization. After 45 min of stabilization time, the polymerization process was completed and an environmentally friendly emulsion with a high water content was obtained. Ex situ systems were prepared by mixing the prepared neat PA emulsion and the TiO_2_ nanoparticles for 2 min on an ultrasonic probe and then for 1 h on a magnetic stirrer.

In situ emulsion polymerization was carried out in the presence of TiO_2_ nanoparticles. An aqueous dispersion of TiO_2_ was prepared and homogenized on an ultrasonic probe for 2 min and then added to the reactor. When 82 °C was reached, the pre-emulsion was added dropwise to the reactor, and a small amount of the APS solution was added at the end to complete the polymerization. After 45 min of stabilization time, the polymerization was completed.

The coatings were prepared on a glass plate using an applicator. The thickness of the wet coatings was 300 µm. The coatings were then dried at room temperature for 24 h followed by drying at 50 ± 5 °C for 24 h. The thickness of the prepared coatings was 50 ± 10 µm.

### 2.3. Accelerated Aging Test

Accelerated aging test of neat PA and PA/TiO_2_ nanocoatings was performed in a Atlas SUNTEST CPS UV chamber (Linsengericht, Germany) equipped with a xenon light source filtered for 290 nm, and irradiance was 1.75 W/(m^2^ nm). The exposure chamber was 20 cm wide × 45 cm long and the coating surface was at 15 cm from the source. The temperature in the UV chamber was approximately 40 °C. The coating films were exposed to UV irradiation and analyzed after 24, 72 and 144 h by differential scanning calorimetry (DSC), IR spectroscopy and gel permeation chromatography (GPC).

### 2.4. Characterization

#### 2.4.1. Particle Size Distribution of Emulsions

The particle size distribution and hydrodynamic diameter of the polyacrylate/TiO_2_ emulsions were studied by dynamic light scattering (DLS) using Malvern Zetasizer Nano device ZS (Malvern, UK). Cell DTS 0012 was used for the dynamic light scattering measurements. The intensity of the scattered light was measured at an angle of 173°. Before measuring particle size distribution, emulsions were homogenized on a magnetic stirrer for 15 min. The particle size was determined from the intensity distribution and the results are presented as the average values of six measurements.

#### 2.4.2. Rheology of Emulsions

Rheological measurements were studied using a rotational rheometer Anton Paar Rheolab QC (Graz, Austria). Before measuring viscosity, emulsions were homogenized on a magnetic stirrer for 15 min. The rheological measurements were performed at room temperature in two steps, first by increasing the shear rate from 0 to 196 s^−1^ and then by decreasing the shear rate from 196 to 0 s^−1^.

#### 2.4.3. Morphology

The morphology of coating films before UV exposure was examined using an Axio Zoom V16 light microscope (Oberkochen, Germany) at 100× magnification on a black background. Scanning electron microscopic (SEM) analysis was performed in high vacuum using a FEI QUANTA 250 FEG scanning electron microscope (Hillsboro, OR, USA) with 10–20 kV accelerating voltage of the electron beam at different magnifications. Both secondary electron and backscattered electron detectors were used for closer observation of differences in morphology and chemical composition. For better imaging and higher resolution in HiVac SEM, the samples were sputtered with a thin layer of Pt/Pd for 30 s.

#### 2.4.4. UV-Vis Transmittance

The transmittance of coating films was measured using a SHIMADZU UV-1650PC UV-Vis spectrophotometer (Columbia, MD, USA) in the range 200–800 nm. The edges of the coating films were adhered to the sanded side of the cuvette so that the test area of the film was on the unsanded side of the cuvette. Before adhering the coating films on the cuvette, the transmittance of the cuvette was measured to subtract it from the transmittance of the coating films/cuvette.

#### 2.4.5. Thermal Properties

Thermogravimetric analysis (TGA) of coating films was performed using TA Instruments Q500 (New Castle, DE, USA). The mass of the sample was ∼10 mg and the heating rate was 10 °C/min from room temperature to 600.0 °C under nitrogen atmosphere.

The glass transition temperature before and after UV exposure of the coating films was determined using differential scanning calorimeter DCS 823 Mettler Toledo (Greifensee, Switzerland) with an intercooler cooling system. Samples used for DSC measurements were weighed (approximately 10 mg) and hermetically sealed in aluminum pans (40 µL). Nitrogen flow of 50 mL min^−1^ was applied throughout the experiments. Samples were cooled to −30 °C and then analysis was started by heating to 70 °C with a thermal sampling rate of 10°C min^−1^ in one cooling and two heating cycles. The reported glass transition temperature was determined from the second heating cycle.

#### 2.4.6. IR Analysis

Chemical changes in the coating films during UV exposure were analyzed using a PerkinElmer Spectrum One spectrometer (Waltham, MA, USA). All coating films were recorded in horizontal micro-ATR mode using Diamant Durascope ATR accessories in the frequency range of 4000 cm^−1^ to 650 cm^−1^ at room temperature. To increase the signal-to-noise ratio, each coating film was scanned four times with a resolution of 4 cm^−1^. The measurements were performed before and after UV exposure.

#### 2.4.7. Molecular Weight Distribution

Changes in molecular weight distribution of coating films during UV exposure were determined by gel permeation chromatography (GPC). Measurements were performed on a chromatography instrument PL-GPC 20 Polymer Laboratories (Santa Clara, CA, USA) equipped with a refractometric sensor. The distribution unit consists of two PLgelMixed-B columns connected in series and filled with poly (styrene/divinylbenzene) terpolymer gel of particle size 3–100 μm and tetrahydrofuran as solvent. Polystyrene standard was used for calibration. One hundred and fifty microliter aliquots of samples were injected into the system and analysis was performed for 25 min.

## 3. Results and Discussion

### 3.1. Particle Size Distribution and Viscosity of Emulsions

**Figure 2:**
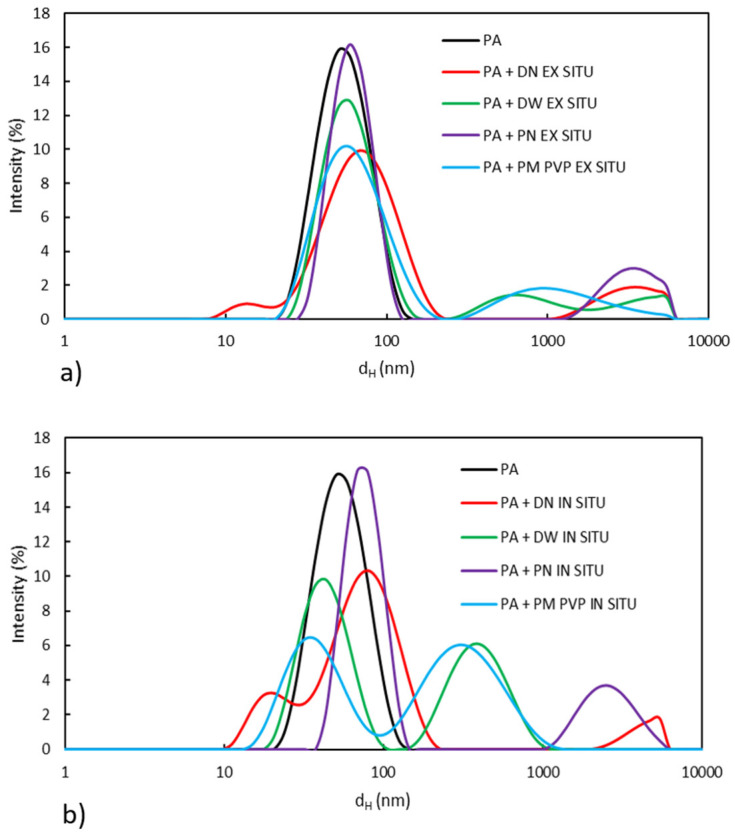
Particle size distribution of neat PA and (**a**) ex situ and (**b**) in situ PA/TiO_2_ emulsions.

To investigate the influence of different types of TiO_2_ nanofillers in polyacrylate emulsion, particle size distribution and viscosity were measured. Changes in particle size distribution after the addition of TiO_2_ in polyacrylate emulsion are visible in Figure 2a,b.

The particle size distribution curve for pure PA is monomodal. The maximum peak of particle size distribution is at a hydrodynamic diameter of 57 nm, which is in accordance with the theory [26] that the emulsion particle size is in the range of 50–300 nm. The addition of TiO_2_ nanofillers affects the particle size distribution of PA, so that ex situ and in situ-prepared PA/TiO_2_ emulsions show a bimodal and in some emulsions a multimodal particle size distribution. The observed differences could be related to the agglomeration of TiO_2_, but also to different mechanisms of nucleation in the presence of TiO_2_ nanoparticles in the process of emulsion polymerization [27,28,29]. Namely, during in situ emulsion polymerization, SDS molecules are adsorbed on the surface of TiO_2_ by electrostatic interactions and form TiO_2_–SDS micelles, into which monomer molecules then enter. Therefore, the nucleation sites in in situ preparation are also TiO_2_–SDS micelles. Another approach [30] also suggests that during in situ emulsion polymerization, the adsorption of the APS initiator occurs on the TiO_2_ surface and polymerization is initiated from the TiO_2_ surface. These early findings [27,28,29,30] indicate that PA is formed around the TiO_2_ nanoparticles during in situ preparation. The mentioned fact and the agglomeration of TiO_2_ particles lead to differences in the particle size distribution of the nanocomposites compared to neat PA. According to Figure 2b, in situ emulsions (except with PN nanofiller) have a smaller amount of particles above 1000 nm, which is due to a lower concentration of agglomerates compared to emulsions prepared ex situ. Emulsions with DW and PM PVP nanoparticles prepared by in situ polymerization have the largest peak around 400 nm, which is significantly smaller compared to other prepared emulsions (Figure 2a,b).

**Figure 3:**
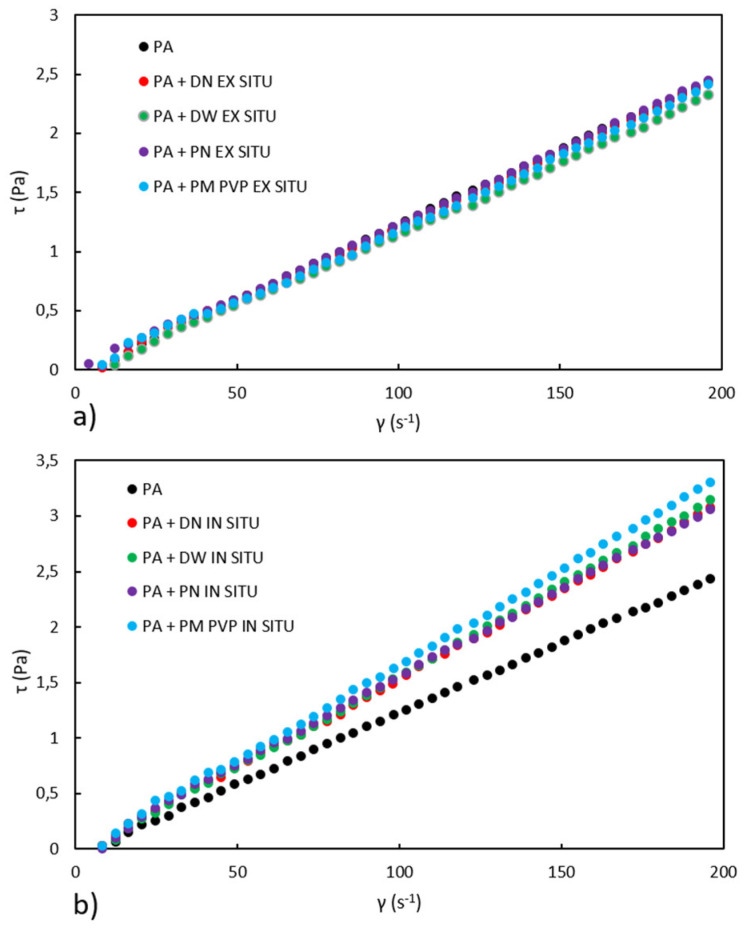
Rheological curves for the PA and (**a**) ex situ and (**b**) in situ PA/TiO_2_ emulsions.

Many factors affect the rheology of emulsions, such as interparticle interactions, interfacial properties, particle size, size distribution, solid particle concentration, emulsion preparation processes, and storage conditions [31]. For the same concentration of solid particles of different sizes, the viscosity can be different depending on the particle size and particle size distribution [31]. Figure 3a,b show the curves of shear stress (τ) versus shear rate (γ), while the viscosities of ex situ and in situ PA/TiO_2_ emulsions are shown in Table 2.

According to Figure 3a,b, a linear relationship between shear stress and shear rate is evident, indicating that all emulsions exhibit Newtonian behavior, i.e., viscosity does not depend on shear rate. The occurrence of rheopexy, an increase in the viscosity of the emulsion over time at a constant shear rate, is not observed in any prepared emulsion. Previous studies [32] of PA with a higher proportion of SiO_2_ nanofillers (≥15 wt%) have indicated the occurrence of rheopexy. Ex situ emulsions show a very similar rheological curve as neat PA emulsion, from which it can be concluded that the addition of nanofillers in ex situ-prepared emulsions does not affect the viscosity of PA despite the differences in particle size distribution. The presence of nanofillers in in situ-prepared emulsions affects the rheological properties of the PA emulsion. As mentioned earlier, interactions between particles have a significant effect on viscosity, and changes in viscosity in in situ-prepared emulsions are not easily explained. However, it is likely that in situ-prepared emulsions have better interactions between PA and TiO_2_ than ex situ-prepared emulsions. Therefore, the increase in viscosity in in situ-prepared emulsions can be attributed to significant interactions between TiO_2_ and PA that produce higher flow resistance and hence higher viscosity values compared to ex situ-prepared emulsions.

### 3.2. Morphology Analysis

**Figure 4:**
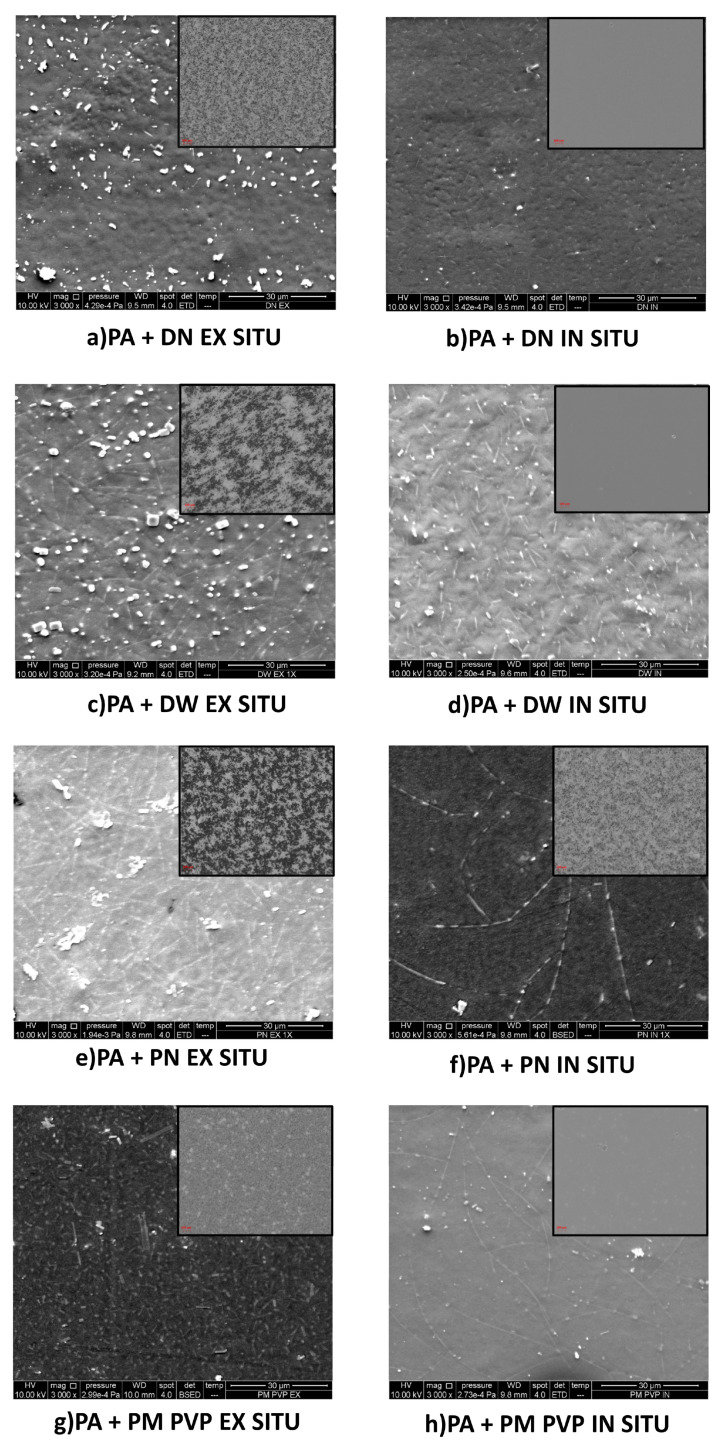
SEM micrographs (main picture) and optical micrographs (inserted picture) of PA/TiO_2_ nanocoatings; (**a**) PA + DN EX SITU, (**b**) PA + DN IN SITU, (**c**) PA + DW EX SITU, (**d**) PA + DW IN SITU, (**e**) PA + PN EX SITU, (**f**) PA + PN IN SITU, (**g**) PA+ PM PVP EX SITU, (**h**) PA + PM PVP IN SITU.

Optical and SEM micrographs of PA and PA/TiO_2_ coating films are shown in Figure 4. TiO_2_ nanoparticles tend to agglomerate in the polymer due to their higher surface energy [33,34,35] and incompatibility with the polymer matrix. Coating films with nanofillers in aqueous dispersions DN and DW show a better distribution of nanofillers in emulsions prepared in situ compared to the emulsions prepared ex situ. The morphologies of the ex situ and in situ coating films with DN filler show the presence of agglomerates, which was confirmed by the particle size distribution results. However, the in situ coating film shows a lower amount of agglomerated TiO_2_ particles compared to the ex situ coating. Additionally, in the coating film with DW filler, the distribution of nanoparticles is better in the in situ-prepared coating compared to the ex situ coating. On the particle size distribution curve of PA + DW in situ emulsion, particles above 1000 nm are not present (Figure 2b), which explains the low amount of agglomerated TiO_2_ particles in the in situ-prepared coating film with DW.

The morphology of the in situ coating film with PN is also better compared to the ex situ coating film. The surface of the PM PVP nanofiller is modified with polyvinylpyrrolidone (PVP), whose role is to increase the stability of the TiO_2_ [36]. The surface coverage of TiO_2_ with a polymer layer of PVP provides efficient repulsion between TiO_2_ particles in aqueous suspensions [37], so that better distribution and better dispersion of this nanofiller in PA can be expected. The ex situ-prepared coating film with PM PVP nanofiller shows more uniform dispersion compared to the ex situ coating film with unmodified PN filler, confirming the effectiveness of TiO_2_ modification with PVP. The in situ coating film with PM PVP also exhibits good dispersion of the nanofiller—significantly better than the in situ coating film with PN filler. The morphology of the in situ coating film with PM PVP is better compared to the ex situ coating film with PM PVP. From all the facts, it can be concluded that the preparation method significantly affects the morphology of coating films such that in situ-prepared coatings have better morphology compared to ex situ-prepared coating films. This is in agreement with previous studies, which indicated that better morphology is achieved by the in situ preparation method [38]. Moreover, the surface modification of TiO_2_ with PVP contributes to a better dispersion of the nanofillers in the PA polymer.

**Figure 5:**
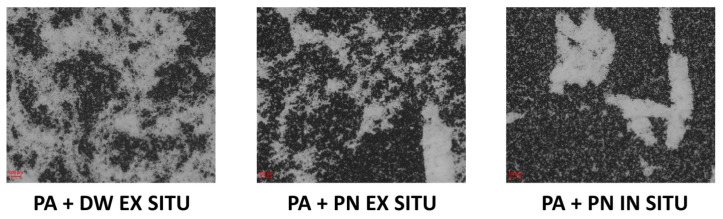
Optical micrographs of PA/TiO_2_ coatings after 30 days.

Regarding the stability of the investigated coating films stored under normal conditions, a subsequent agglomeration of the nanofillers was observed in some emulsions. Therefore, after 30 days, the coating films from all emulsions were prepared again and significant agglomeration of nanofillers was observed in the coating films with DW and PN nanofillers prepared ex situ and in the coating with PN nanofiller prepared in situ (Figure 5).

It can be seen that the ex situ coating film with DW has a worse dispersion of the nanofiller compared to the coating before it (Figure 4), while in the coating films with PN prepared by the ex situ and in situ methods, the dispersion of the nanofiller is significantly worse, as large (compact) agglomerates are visible. Additionally, it was found that the agglomerates could not be redispersed by mixing or sonication. For all other emulsions, no instability in the sense of subsequent agglomeration of the nanofiller particles was observed.

### 3.3. UV–Vis Transmittance

**Figure 6:**
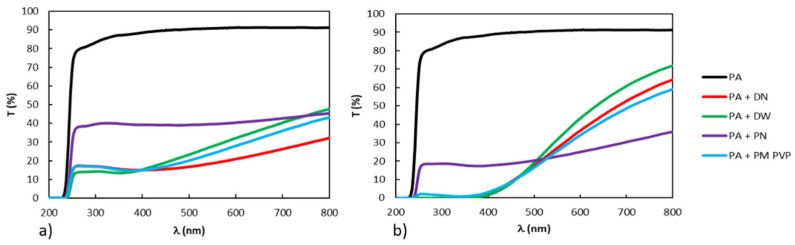
UV–Vis spectra of PA/TiO_2_ (**a**) ex situ and (**b**) in situ-prepared nanocoatings.

Figure 6a,b show the ultraviolet-visible (UV-Vis) transmission spectra for the PA/TiO_2_ coating films prepared by ex situ and in situ methods.

The neat PA film has high transmittance in the UV region, but also in the visible region, indicating the good transparency of the PA film. The addition of TiO_2_ nanofiller significantly reduces the transmittance of the PA coating films in the UV and visible parts of the spectrum. For metal oxides, such as TiO_2_, previous studies indicate that it tends to absorb UV radiation, especially at lower wavelengths [3,13,39]. Comparing the transmittance values of ex situ and in situ-prepared coatings, it is found that in situ-prepared coatings provide better UV protection. Moreover, in situ-prepared coatings (Figure 6b) exhibit higher transmittance in the visible region of the spectrum compared to ex situ-prepared coatings (Figure 6a) and hence better transparency, which can be explained by the better dispersion of TiO_2_ nanofillers in these systems (Figure 4). The coating film that offers the highest transparency in the visible range and the highest UV protection is the in situ coating film with DW filler.

### 3.4. Thermal Stability

In order to evaluate thermal stability, thermogravimetric analysis of the PA and PA/TiO_2_ coating films was performed. Due to the large number of results and the similarity of the DTG and TGA curve shapes, only the TGA/DTG curves of the selected coating films are reported here. All the investigated coating films were analyzed and their results are given in Table 3.

**Figure 7:**
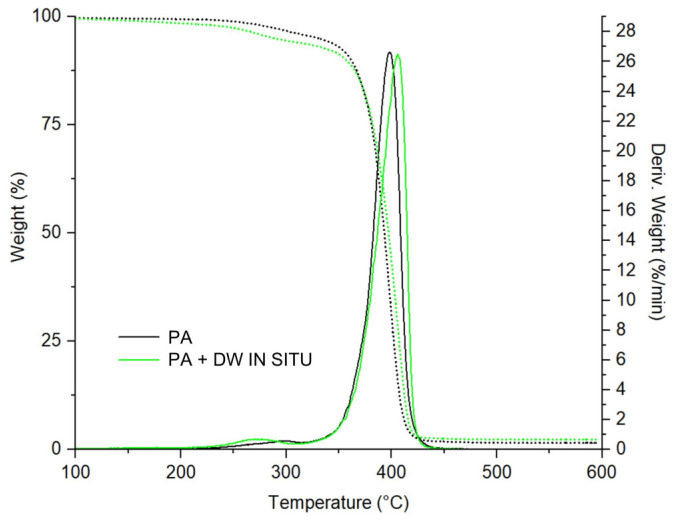
Derivative thermogravimetric (DTG, solid line) and thermogravimetric (TGA, dashed line) curves of neat PA and PA + DW in situ nanocoating films.

The results are represented by the thermal degradation onset temperature (*T*_90_), at which 10% of weight loss occurred, and half degradation temperature (*T*_50_), at which 50% of weight loss occurred. It can be assumed that the temperature at 10% weight loss (*T*_90_) is the initial temperature of thermal degradation. Figure 7 shows the DTG and TGA curves of neat PA and the PA + DW in situ coating film.

The DTG curves of PA and PA/TiO_2_ coating films show two stages of thermal degradation, the first in the range of 230 to 320 °C and the second, larger, in the range of 350 to 450 °C. The first stage of thermal degradation is mainly due to the removal of volatile unreacted monomers or emulsifier residues [40]. Furthermore, in the second stage of degradation at a temperature above 350 °C, a pronounced degradation step with the highest weight loss caused by random chain scission [41] was observed in all tested coating films. The thermal degradation onset temperature (*T*_90_) is lower for all PA/TiO_2_ coating films compared to pure PA, indicating that the addition of TiO_2_ nanofillers decreases the thermal stability of PA at the start of thermal degradation. The weight loss of the nanocoatings in the early stage of thermal degradation is due to the hydroxyl groups on the TiO_2_ surface, which catalyze the thermal degradation of PA [42]. The half degradation temperature, *T*_50_, of PA/TiO_2_ coating films is not significantly different from that of PA, indicating that the addition of TiO_2_ nanofillers does not affect the thermal stability of PA in the later stages of degradation. A possible explanation for the obtained results is reduced interactions between the BA segment in the PA polymer and TiO_2_ at high temperatures [41]. BA has a very low glass transition temperature (−49 to −53 °C), and therefore it cannot properly bond with TiO_2_ at high temperatures. The interfacial bonding between the BA segment in the polymer and the TiO_2_ nanoparticles may become weaker at high temperatures, leading to a weakening of the thermal stability of PA by the addition of TiO_2_ nanofillers [41]. From the thermal stability results, it can be concluded that the addition of TiO_2_ nanofillers does not improve the thermal stability; on the contrary, at the beginning of degradation, it even reduces the thermal stability of PA. In contrast to the results of this work, some previous studies [20,21,43,44] report a faster degradation rate for the neat PA than for the PA/TiO_2_ coating films and attribute this to the fact that the TiO_2_ particles act as thermal barriers in the early stages of thermal degradation and consequently improve the thermal stability of the coating films.

### 3.5. Glass Transition Temperature

**Figure 8:**
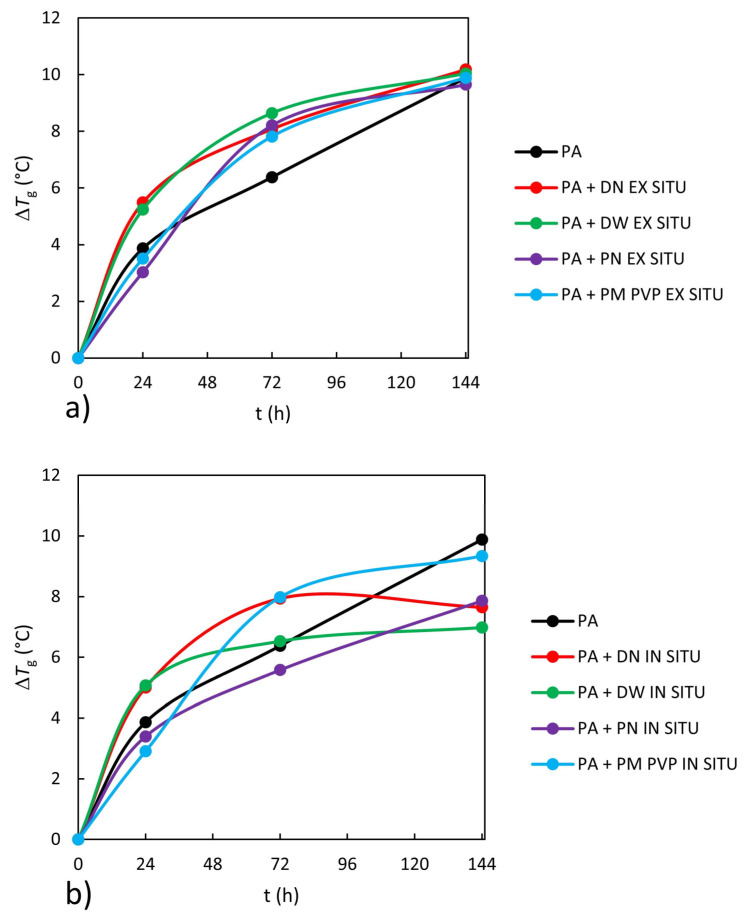
Changes in Δ*T*_g_ for neat PA and PA/TiO_2_ (**a**) ex situ and (**b**) in situ nanocoating films during UV exposure.

The difference between the *T*_g_ values after and before a certain time of UV exposure (Δ*T*_g_) for neat PA and PA/TiO_2_ nanocoating films is shown in Figure 8a,b. According to Figure 8, UV exposure increases the glass transition temperature of neat PA and PA/TiO_2_ coating films. This trend of *T*_g_ increase by UV exposure is attributed to the formation of cross-linked structures since cross-linking further restricts the segmental motion of macromolecules and reduces the mobility and free volume of polymer chains [45,46]. It was interesting to observe that coating films prepared ex situ showed a higher increase in glass transition temperatures compared to neat PA. Previous studies [3,25] have also shown that after UV exposure PA coatings with TiO_2_ exhibit a higher increase in glass transition temperature than neat PA, which was attributed to the photocatalytic effect of TiO_2_ nanoparticles. In situ-prepared coating films with powder (PN and PM PVP) as nanofillers have a slight effect on the glass transition temperature at the beginning of UV degradation (after 24 h UV exposure), while the coatings with nanofillers in water dispersions (DN and DW) show an increase in glass transition temperature compared to neat PA. However, after further UV exposure up to 144 h, all the investigated PA/TiO_2_ in situ-prepared coating films show less change in glass transition temperature compared to neat PA, indicating the UV-protective effect of TiO_2_. UV absorption of TiO_2_ nanoparticles can produce two opposing effects: UV protection or UV degradation of polymers due to the photocatalytic activity of TiO_2_. The photocatalytic effect arises from the possibility of the formation of pairs of positive electron holes, which together with negative electrons can react with water, oxygen molecules or hydroxyl groups on the surface of TiO_2_ nanoparticles, leading to the formation of free radicals. These free radicals are responsible for the degradation of polymers by initiating photocatalytic reactions.

The results indicate that the method of preparation affects the UV protection properties of the coating film, thus ex situ coating films show no UV protection properties, while in situ coating films show better UV protection. The morphology results comparatively showed higher agglomeration in ex situ coating films as compared to in situ-prepared coating films. Agglomerated TiO_2_ nanoparticles with hydroxyl groups form water-rich regions with the polymer PA, which induce UV degradation [13]. Additionally, the UV–Vis transmittance results showed lower transmittance in the UV region of the in situ coating films compared to ex situ (Figure 6a,b).

### 3.6. Chemical Changes

**Figure 9:**
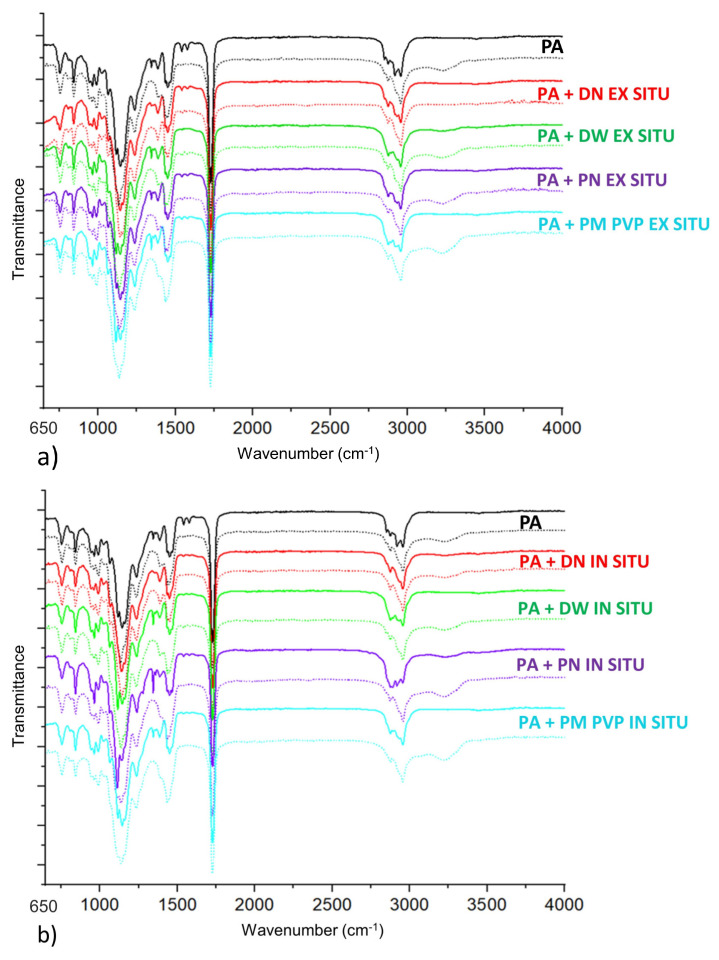
FTIR spectra of neat PA and PA/TiO_2_ ex situ (**a**) and in situ (**b**) coating films, before (solid line) and after 144 h UV exposure (dashed line).

Infrared spectroscopy is a sensitive and reliable technique that allows the quantification of chemical changes caused by a UV degradation process. The UV degradation process of coating films involves photooxidation processes of the polymer matrix, such as chain scission, cross-linking and the formation of oxidation products. In addition, polymer photodegradation may be due to chain scission and macroradical disproportionation, resulting in terminal double bonds [13]. In systems containing TiO_2_, this macroradical disproportionation can be generated due to TiO_2_ as a photocatalyst exposed to UV radiation. This macroradical disproportionation would attack polymer chains to generate –OH and C=O groups [13,22,47]. The FTIR spectra of the coating films are shown in Figure 9a,b. The obtained spectra of all samples show bands that are attributed to polyacrylates, and they agree well with the literature data [48,49]. The bands visible in the spectrum in the range 2956–2873 cm^−1^ correspond to the C–H stretching of the methyl (–CH_3_) and methylene (–CH_2_) groups. The particularly pronounced band at 1727 cm^−1^ indicates the carbonyl stretching vibration, C=O. Furthermore, the bands at 1236 cm^−1^ correspond to C–C–O bending, at 1140 cm^−1^ to C–O–C bending and at 755 cm^−1^ to C–C group bending. Due to the addition of TiO_2_ nanofillers, bands related to the stretching of Ti–O bands and the bending vibration of Ti–OH bands are expected to be in the range of 400–800 cm^−1^ and at 1400 cm^−1^, respectively. The aforementioned bands were not observed, indicating that the TiO_2_ band is covered by PA bands in all the studied coating films. However, it is important to note that the TiO_2_ concentration in the prepared coating films was only 1 wt% and because of that low concentration may be below the detection limits of the FTIR instrument [50].

From the obtained FTIR spectrum (Figure 9a,b) of coating films before (solid line) and after 144 h of UV exposure (dashed line), it is evident that after UV exposure the –OH band appears, which can be associated with the oxidation reactions that occur during UV degradation [51,52,53,54]. Additionally, the changes in the 2956 cm^−1^ and 2873 cm^−1^ bands, which are also visible, may indicate the formation of monomers due to the chain scission of PA [3]. Some of the characteristic IR bands of the functional groups of the coating films and their changes after UV exposure are shown in Table 4.

By comparing the intensity of the characteristic bands of the unexposed and exposed coatings, it can be seen from Table 4 that the intensity bands assigned to the methylene group (–CH_2_) at 2873 cm^−1^ decrease and the bands assigned to the methyl group (–CH_3_) at 2956 cm^−1^ increase during UV exposure, which may be related to the formation of monomers due to chain scission, as mentioned earlier. IR bands corresponding to a hydroxyl group (–OH) and a carbonyl group (C=O) are usually increased, which can be related to the formation of oxidation products during the UV degradation process [13,22,47].

The formation of oxidation products –OH and C=O during the accelerated aging process is more pronounced in some PA/TiO_2_ coating films than in the neat PA. In addition, it can be seen that the mechanisms of UV degradation differ depending on the type of TiO_2_ nanofiller. Coating films with nanofillers in powder form have a more pronounced –OH peak than the coating films with nanofillers in aqueous dispersion, indicating the formation of more oxidative products in these coatings than in coatings with nanofillers in aqueous dispersion.

### 3.7. Molecular Weight Distribution

**Figure 10:**
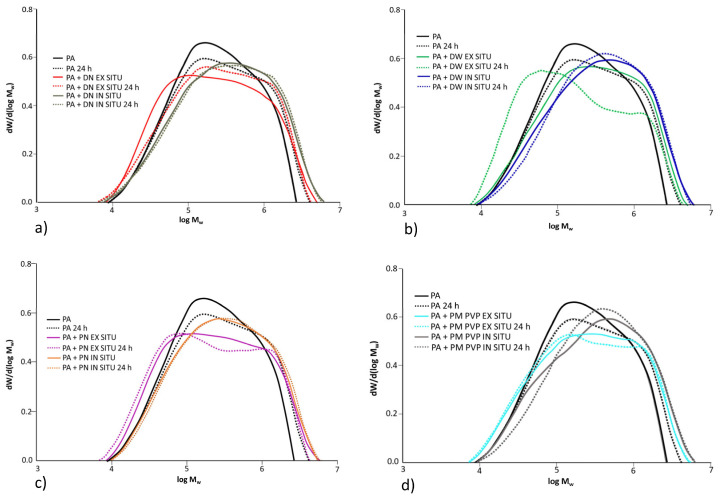
Differential molecular weight distribution curve for neat PA and ex situ and in situ coating films before (solid line) and after UV exposure (dash line); (**a**) PA, PA + DN EX SITU and PA + DN IN SITU before and after UV exposure, (**b**) PA, PA + DW EX SITU and PA + DW IN SITU before and after UV exposure, (**c**) PA, PA + PN EX SITU and PA + PN IN SITU before and after UV exposure, (**d**) PA, PA + PM PVP EX SITU and PA + PM PVP IN SITU before and after UV exposure.

To determine the change in the molecular weight distribution of PA and PA/TiO_2_ coating films due to UV exposure, GPC analysis was performed. GPC analysis was used to study the changes in polymer chain length during photolytic and/or cross-linking reactions at different UV exposure times. Before GPC analysis, the nanocoatings should be dissolved in THF as a solvent. However, the coating films after 72 and 144 h of UV exposure could not be dissolved in THF, indicating the formation of polymer cross-links. Methacrylate polymers are generally less prone to cross-linking due to the higher stability of the tertiary macroradicals formed after chain scission [55,56]. Therefore, it can be suggested that cross-linking primarily affects the butyl acrylate groups in BA/MMA copolymers. The higher mobility of the butyl side chain easily led to decomposition to form alkoxy radicals, which formed the cross-linking. Differential molecular weight distribution curves for neat PA and PA/TiO_2_ ex situ and in situ coating films before and after 24 h of UV exposure are shown in Figure 10.

According to the molecular weight distribution for neat PA, it can be seen that the curve moves towards a larger molecular weight, indicating cross-linking in PA after UV exposure. From the plotted results, it is evident that UV irradiation has a greater effect on changing the molecular weight distribution in ex situ coating films than in situ. The molecular weight distribution for in situ coating films is almost unchanged, and small changes are visible in in situ coating film with PM PVP nanofiller. Ex situ coating films show changes in the shape of the distribution curves, respectively, as peaks move towards a smaller molecular weight, indicating chain scission and macroradical disproportionation. It is interesting to note that the mechanism of UV degradation varies depending on the preparation method. Overall, the results of the changes in glass transition temperature due to UV exposure indicate that the photocatalytic effect of TiO_2_ is more pronounced in the ex situ coating films, while a better UV-protective effect is established for the in situ coating films.

Therefore, a possible reason for the shift towards a smaller molecular weight distribution for ex situ coating films is the chain scission in the earlier stages of UV degradation, while in situ coating films do not show significant changes in molecular weight distribution upon UV exposure, confirming the UV-protective properties.

## 4. Conclusions

Environmentally friendly PA/TiO_2_ emulsions with various types of TiO_2_ nanofillers have been successfully prepared by ex situ and in situ emulsion polymerization. The addition of TiO_2_ nanoparticles significantly affects the particle size distribution of PA, and in situ emulsions have smaller agglomerates compared to ex situ emulsions. The viscosity of the emulsions changes depending on the preparation method, so for in situ emulsions there is an increase in viscosity with the addition of TiO_2_ nanofillers. The morphology differs significantly with respect to the preparation method and the addition of nanofillers, so in situ coating films with nanofillers in water dispersion have the best morphology. Moreover, in situ coating films with nanofillers in water dispersion significantly reduce the transmission of UV light and have the best transparency. The glass transition temperature increases upon UV exposure for all coating films, indicating the cross-linking in the polymer due to UV radiation, but this increase is less pronounced for in situ coating films compared to neat PA; therefore, in situ coating films provide a better UV protection effect. TiO_2_ in ex situ coating films has a pronounced photocatalytic effect and accelerates the process of the UV degradation of PA, which is probably due to the poor dispersity of the nanofiller. Taking into account the use of these coating films as UV protectives for wood surfaces, the moderate increase in *T*_g_ values, remaining within the acceptable range of use, i.e., below room temperature, indicates that the necessary film plasticity is maintained. Moreover, the changes in the molecular weight distribution were more pronounced in the ex situ coating films than in the in situ films. This work provides some useful findings about the significant influence of the preparation method on the properties of PA/TiO_2_ coating films. Further improvements in the effect of TiO_2_ on the UV-protective properties and transparency of PA coatings can be achieved by optimizing the amount of TiO_2_ nanofiller. Since this study showed the better properties and UV protection effect of TiO_2_ nanofiller in the form of aqueous dispersion, further research will focus on studying the effect of its amount on the UV protection properties of PA nanocoatings.

## Figures and Tables

**Table 1:** The properties of TiO_2_ nanofillers.

Label of Nanofiller	
DN	TiO_2_, 40 wt% aqueous dispersion, rutile form, average particle size 30 nm, particle size range 10–120 nm
DW	TiO_2_, 20 wt% aqueous dispersion, fully dispersed in water, rutile form, average particle size 30 nm, particle size range 15–80 nm
PN	TiO_2_, nanopowder, rutile form, high purity 99.9%, average particle size 30 nm
PM PVP	TiO_2_, nanopowder, rutile form, high purity 99.9%, average particle size 30 nm, modified with 1–2 wt% PVP (polyvinyl pyrrolidone)

**Table 2:** Values of viscosity *η* for PA and PA/TiO_2_ ex situ and in situ emulsions.

Type of Coating	*η* (mPa×s)	
**PA**	12.8	
	**ex situ**	**in situ**
PA + DN	12.7	16.0
PA + DW	12.1	16.6
PA + PN	12.5	16.1
PA + PM PVP	12.3	17.2

**Table 3:** Thermal degradation onset temperature (*T*_90_) and half degradation temperature (*T*_50_) for neat PA and PA/TiO_2_ ex situ and in situ nanocoating films.

Type of Coating Films	*T*_90_ (°C)		*T*_50_ (°C)	
**PA**	361.6		393.2	
	**ex situ**	**in situ**	**ex situ**	**in situ**
PA + DN	358.7	355.3	396.4	391.7
PA + DW	358.2	353.9	393.6	394.1
PA + PN	354.9	351.1	390.1	389.1
PA + PM PVP	256.2	357.5	393.2	390.6

**Table 4:** IR absorption characteristic changes of functional groups in PA and PA/TiO_2_ coating films before and after 144 h UV exposure.

IR Band (cm^−1^)	Functional Group	Before UV Exposure	After 144 h of UV Exposure	Observations	
				Intensity	Changes
3228	O–H	-	+	weak	increased
2956	C–H stretching of the methyl (–CH_3_) groups	+	+	strong	increased
2873	C–H stretching of methylene (–CH_2_) groups	+	+	weak	decreased
1726	C=O stretching in ester group	+	+	very strong	increased

Note: “+”: absorption; “-”: no absorption.

## Data Availability

The data presented in this study are available on request from the corresponding author.

## References

[1] Nuopponen M., Wikberg H., Vuorinen T., Maunu S., Jamsa S., Viitaniemi P. (2003). Heat-treated softwood exposed to weathering. J. Appl. Polym. Sci..

[2] Hollosy F. (2002). Effects of ultraviolet radiation on planet cells. Micron.

[3] Miklečić J., Blagojević S.L., Petrič M., Jirouš-Rajković V. (2015). Influence of TiO_2_ and ZnO nanoparticles on properties of waterborne polyacrylate coating exposed to outdoor conditions. Prog. Org. Coat..

[4] Decker C., Ebnesajjd S. (2011). UV-Radiation Curing of Adhesives. Handbook of Adhesives and Surface Preparation.

[5] Rabek J. (1995). Polymer Photodegradation.

[6] Soeriyadi A., Trouillet V., Bennet F., Burns M., Whittaker M., Boyer C., Barker P., Davis T., Barner-Kowolik C. (2012). A Detailed Surface Analytical Study of Degradation Processes in (Meth) acrylic Polymers. J. Polym. Sci. Part A Polym. Chem..

[7] Jirouš-Rajković V., Miklečić J. (2021). Enhancing Weathering Resistance of Wood—A Review. Polymers.

[8] Bao Y., Shi C., Ma J., Wang B., Zhang Y. (2015). Double in-situ synthesis of polyacrylate/nano TiO_2_ composites latex. Prog. Org. Coat..

[9] Godnjavec J., Znoj B., Vince J., Steinbucher M., Žnidaršić A., Venturini P. (2012). Stabilization of rutile TiO_2_ nanoparticles with glymo in polyacrylic clear coating. Mater. Tehnol..

[10] Allena N., Edgea M., Ortegaa A., Liauwa C., Strattonb J., McIntyreb R. (2002). Behaviour of nanoparticle (ultrafine) titanium dioxide pigments and stabilisers on the photooxidative stability of water based acrylic and isocyanate based acrylic coatings. Polym. Degrad. Stab..

[11] Ye C., Li H., Cai A., Zeng X. (2011). Preparation and characterization of organic nano-titanium dioxide/acrylate composites emulsions by in-situ emulsion polymerization. J. Macromol. Sci. Part A Pure Apple. Chem..

[12] Dahapte V., Gaikwad N., More P.V., Banerjee S., Dhapte V.V., Kadam S., Khanna P. (2015). Transparent ZnO/polycarbonate nanocomposite for food packaging application. Nanocomposites.

[13] Nguyen T., Dao P.H., Doung K.L., Doung Q.H., Vu Q.T., Nguyen H., Phuc Mac V., Le T.L. (2017). Effect of R-TiO_2_ and ZnO nanoparticles on the UV-shielding efficiency of water-borne acrylic coating. Prog. Org. Coat..

[14] Nikolic M., Lawther J.M., Sanadi A.R. (2015). Use of nanofillers in wood coatings: A scientific review. J. Coat. Technol. Res..

[15] Godnjavec J., Znoj B., Venturini P., Žnidaršič A. (2010). The application of rutile nano-crystalline titanium dioxide as UV absorber. Inf. MIDEM.

[16] Noman M.T., Ashraf M.A., Jamshaid H., Ali A. (2018). A Novel Green Stabilization of TiO_2_ Nanoparticles onto Cotton. Fibers Polym..

[17] Noman M.T., Militky J., Wiener J., Saskova J., Ashraf M.A., Jamshaid H., Azeem M. (2018). Sonochemical synthesis of highly crystalline photocatalyst for industrial applications. Ultrasonics.

[18] Ugur S., Sarııšık S., Aktaş H. (2011). Nano-TiO_2_ based multilayer film deposition on cotton fabrics for UV-protection. Fibers Polym..

[19] Veronovski N., Verhovšek D., Godnjavec J. (2013). The influence of surface-treated nano-TiO_2_ (rutile) incorporation in water-based coatings on wood protection. Wood Sci. Technol..

[20] Wang C., Sheng X., Xie D., Zhang X., Zhang H. (2016). High-performance TiO_2_/polyacrylate nanocomposites with enhanced thermal and excellent UV-shielding properties. Prog. Org. Coat..

[21] Man Y., Mu L., Wang Y., Lin S., Rempel G., Pan Q. (2014). Synthesis and characterization of rutile titanium dioxide/polyacrylate nanocomposites for applications in ultraviolet light-shielding materials. Polym. Compos..

[22] Nguyen T., Tri P.N., Nguyen T., Elaidani R., Trinh V., Decker C. (2016). Accelerated degradation of water borne acrylic nanocomposites used in outdoor protective coatings. Polym. Degrad. Stab..

[23] Antunes A., Popelka A., Aljarod O., Hassan M.K., Kasak P., Luyt A. (2020). Accelerated weathering effects on poly (3-hydroxybutyrate-co-3-hydroxyvalerate) (PHBV) and PHBV/TiO_2_ nanocomposites. Polymers.

[24] Yang T., Noguchi T., Isshiki M., Wu J. (2014). Effect of titanium dioxide on chemical and molecular changes in PVC sidings during QUV accelerated weathering. Polym. Degrad. Stab..

[25] Aloui F., Ahajji A., Irmouli Y., George B., Charrier B., Merlin A. (2007). Inorganic UV absorbers for the photostabilisation of wood-clearcoating system: Comparison with organic UV absorbers. Appl. Surf. Sci..

[26] Odian G. (2004). Principles of Polymerization.

[27] Zhang J., Zheng P., Sun X., Wang C., Gao J. (2010). Synthesis and kinetic studies of TiO_2_/polystyrene composite particles by emulsion polymerization. e-Polymers.

[28] Bourgeat-Lami E., Duguet E., Ghosh S.K. (2006). Polymer Encapsulation of Inorganic Particles. Functional Coatings: By Polymer Microencapsulation.

[29] Bourgeat-Lami E., Kickelbick G. (2006). Hybrid Organic/Inorganic Particles. Hybrid Materials: Synthesis, Characterization, and Applications.

[30] Luna-Xavier J., Bourgeat-Lami E., Guyot A. (2001). The role of initiation in the synthesis of silica/poly (methyl methacrylate) nanocomposite latex particles through emulsion polymerization. Colloid Polym. Sci..

[31] Schneider M., Claverie J., McKenna A. (2002). High solids content emulsions. I. A study of the influence of the particles size distributions and polymer concentration on viscosity. J. Appl. Polym. Sci..

[32] Buhin Z. (2013). Emulzijska In Situ Polimerizacija i Karakterizacija poli [(butil-akrilat)-co-metilmetakrilat)]/Silika Nanosustava. Doctoral Thesis.

[33] Domingos R., Tufenkji N., Wilkinson K. (2009). Aggregation of titanium dioxide nanoparticles: Role of a fulvic acid. Environ. Sci. Technol..

[34] French R., Jacobson A., Kim B., Isley S., Penn R., Baveye P. (2009). Influence of ionic strength, pH, and cation valence on aggregation kinetics of titanium dioxide nanoparticles. Environ. Sci. Technol..

[35] Guzman K.D., Finnegan M., Banfield J. (2006). Influence of surface potential on aggregation and transport of titania nanoparticles. Environ. Sci. Technol..

[36] Du B., Chen F., Luo R., Zhou S., Wu Z., Li J. (2019). Investigation on the coating modification effect of nano-SiO_2_ particles on nano-TiO_2_ particles. Mater. Res. Express.

[37] Koczkur K., Mourdikoudis S., Polavarapu L., Skrabalak S. (2015). Polyvinylpyrrolidone (PVP) in nanoparticle synthesis. Dalton Trans..

[38] He Q., Wu L., Gu G., You B. (2002). Preparation and characterization of acrylic/nano-TiO_2_ composite latex. High Perform. Polym..

[39] Blanchard V., Blanchet P. (2011). Color stability for wood products during use: Effects of inorganic nanoparticles. BioResources.

[40] Tao W., Fei F., Wang Y.-C. (2006). Structure and thermal properties of titanium dioxide-polyacrylate nanocomposites. Polym. Bull..

[41] Poddar M.K., Sharma S., Pattipaka S., Pamu D., Moholkar V. (2017). Ultrasound-assisted synthesis of poly (MMA-co-BA)/ZnO nanocomposites with enhanced physical properties. Ultrason. Sonochem..

[42] Ashraf M., Peng W., Zare Y., Rhee K.Y. (2018). Effects of size and aggregation/agglomeration of nanoparticles on the interfacial/interphase properties and tensile strength of polymer nanocomposites. Nanoscale Res. Lett..

[43] Sheng X., Xie D., Wang C., Zhang X., Zhong L. (2016). Synthesis and characterization of core/shell titanium dioxide nanoparticles/polyacrylate nanocomposites colloidal microspheres. Colloid Polym. Sci..

[44] Zhu J., Uhl F.M., Morgan A.B., Wilkie C. (2001). Studies on the mechanism by which the formation of nanocomposites enhances thermal stability. Chem. Mater..

[45] Aloui F., Ahajji A., Irmouli Y., George B., Charrier B., Merlin A. (2006). Photostabilization of the “wood-clearcoatings” systems with UV absorbers: Correlation with their effect on the glass transition temperature. J. Phys. Conf. Ser..

[46] Cristea M., Riedl B., Blanchet P. (2010). Enhancing the performance of exterior waterborne coatings for wood by inorganic nanosized UV absorbers. Prog. Org. Coat..

[47] Shanti R., Hadi A.N., Salim Y.S., Chee S.Y., Ramesh S., Ramesh K. (2017). Degradation of ultra-high molecular weight poly (methyl methacrylate-co-butyl acrylate-co-acrylic acid) under ultra violet irradiation. RSC Adv..

[48] Liufu S., Xiao H.N., Li Y. (2005). Thermal analysis and degradation mechanism of polyacrylate/ZnO nanocomposites. Polym. Degrad. Stab..

[49] Liang D., Du C., Ma F., Shen Y., Wu K., Zhou J. (2018). Degradation of polyacrylate in the outdoor agricultural soil measured by FTIR-PASS and LIBS. Polymers.

[50] Goncalves Mota R.A., da Silva E.O., de Menezes L.R. (2018). Effect of the addition of metal oxide nanoparticles on the physical, chemical and thermal properties of PVA based nanocomposites. Mater. Sci. Appl..

[51] Wochnowski C., Eldin M.A.S., Metev S. (2005). UV-laser-assisted degradation of poly (methyl methacrylate). Polym. Degrad. Stab..

[52] Aguirre M., Goikoetxea M., Otero L.A., Paulis M. (2017). Accelerated ageing of hybrid acrylic waterborne coatings containing metal oxide nanoparticles: Effect on the microstructure. Surf. Coat. Technol..

[53] Del Grosso C., Poulis J., de la Rie E.R. (2019). The photo-stability of acrylic tri-block copolymer blends for the consolidation of cultural heritage. Polym. Degrad. Stab..

[54] Sbardella F., Pronti L., Santarelli M., Asua Gonzalez J., Bracciale M. (2018). Waterborne acrylate-based hybrid coatings with enhanced resistance properties on stone surfaces. Coatings.

[55] Chiantore O., Trossarelli L., Lazzari M. (2000). Photooxidative degradation of acrylic and methacrylic polymers. Polymer.

[56] Lazzari M., Scalarone D., Malucelli G., Chiantore O. (2011). Durability of acrylic films from commercial aqueous dispersion: Glass transition temperature and tensile behavior as indexes of photooxidative degradation. Prog. Org. Coat..

